# Apixaban 5 and 2.5 mg twice-daily versus warfarin for stroke prevention in nonvalvular atrial fibrillation patients: Comparative effectiveness and safety evaluated using a propensity-score-matched approach

**DOI:** 10.1371/journal.pone.0191722

**Published:** 2018-01-26

**Authors:** Xiaoyan Li, Allison Keshishian, Melissa Hamilton, Ruslan Horblyuk, Kiran Gupta, Xuemei Luo, Jack Mardekian, Keith Friend, Anagha Nadkarni, Xianying Pan, Gregory Y. H. Lip, Steve Deitelzweig

**Affiliations:** 1 Worldwide Health Economics and Outcomes Research, Bristol-Myers Squibb Company, Lawrenceville, NJ, United States of America; 2 STATinMED Research, Ann Arbor, MI, United States of America; 3 Formerly of Pfizer, New York, NY, United States of America; 4 US Health Economics and Outcomes Research, Bristol-Myers Squibb Company, Lawrenceville, NJ, United States of America; 5 Pfizer, Inc., Groton, CT, United States of America; 6 Pfizer, Inc., New York, NY, United States of America; 7 Worldwide Medical, Bristol-Myers Squibb Company, Lawrenceville, NJ, United States of America; 8 Center for Observational Research and Data Science, Bristol-Myers Squibb Company, Wallingford, CT, United States of America; 9 Institute of Cardiovascular Sciences, University of Birmingham, UK, Aalborg Thrombosis Research Unit, Department of Clinical Medicine, Aalborg University, Aalborg, Denmark; 10 Ochsner Clinic Foundation, Department of Hospital Medicine, New Orleans, LA, and The University of Queensland School of Medicine, Ochsner Clinical School, New Orleans, LA, United States of America; Institut d'Investigacions Biomediques de Barcelona, SPAIN

## Abstract

Prior real-world studies have shown that apixaban is associated with a reduced risk of stroke/systemic embolism (stroke/SE) and major bleeding versus warfarin. However, few studies evaluated the effectiveness and safety of apixaban according to its dosage, and most studies contained limited numbers of patients prescribed 2.5 mg twice-daily (BID) apixaban. Using pooled data from 4 American claims database sources, baseline characteristics and outcomes for patients prescribed 5 mg BID and 2.5 mg BID apixaban versus warfarin were compared. After 1:1 propensity-score matching, 31,827 5 mg BID apixaban-matched warfarin patients and 6600 2.5 mg BID apixaban-matched warfarin patients were identified. Patients prescribed 2.5 mg BID apixaban were older, had clinically more severe comorbidities, and were more likely to have a history of stroke and bleeding compared with 5 mg BID apixaban patients. Compared with warfarin, 5 mg BID apixaban was associated with a lower risk of stroke/SE (hazard ratio [HR]: 0.70, 95% confidence interval [CI]: 0.60–0.81) and major bleeding (HR: 0.59, 95% CI: 0.53–0.66). Compared with warfarin, 2.5 mg BID apixaban was also associated with a lower risk of stroke/SE (HR: 0.63, 95% CI: 0.49–0.81) and major bleeding (HR: 0.59, 95% CI: 0.49–0.71). In this real-world study, both apixaban doses were assessed in 2 patient groups differing in age and clinical characteristics. Each apixaban dose was associated with a lower risk of stroke/SE and major bleeding compared with warfarin in the distinct population for which it is being prescribed in United States clinical practice.

**Trial registration:** Clinicaltrials.Gov Identifier: NCT03087487.

## Introduction

Vitamin K antagonists such as warfarin have been used as the anticoagulant therapeutic modality for stroke prevention in patients with atrial fibrillation for several decades [1]. More recently, non-vitamin K antagonist oral anticoagulants (NOACs) are being used at greater frequencies and have several advantages to vitamin K antagonists, such as fewer drug–food interactions and no anticoagulation monitoring being required [2]. In phase 3 clinical trials, NOACs have demonstrated at least equivalent efficacy and safety compared to warfarin [3–6]. Apixaban was the only NOAC to show risk reductions in both stroke/systemic embolism (stroke/SE) and major bleeding compared with warfarin in its phase 3 clinical trial [5].

Apixaban is available as: 5 mg twice daily (BID) and 2.5 mg BID. The recommended dose is 2.5 mg BID apixaban if patients meet ≥2 of the following criteria: aged ≥80 years, body weight ≤60kg, and serum creatinine level ≥1.5mg/dL [7]. In the Apixaban for Reduction in Stroke and Other Thromboembolic Events in Atrial Fibrillation (ARISTOTLE) trial, 4.7% of patients in the apixaban group (n = 428) received 2.5 mg BID apixaban, and no significant interaction was observed between dose and treatment effect regarding stroke/SE and major bleeding [5]. A subanalysis of ARISTOTLE trial data by Alexander et al. suggested that the use of apixaban 5 mg BID is appropriate for patients meeting only one of these dose reduction criteria [8]. Although previous real-world studies have shown that apixaban is associated with a reduced risk of stroke/SE and major bleeding versus warfarin, most of these studies contained only limited numbers of patients taking 2.5 mg BID. In addition, few studies have evaluated the effectiveness and safety of apixaban according to dosage, or have taken into consideration patient characteristics related to dose reduction criteria [9–13]. Certain patient characteristics—including older age and renal disease—are associated with an increased risk of stroke and major bleeding, and dose-reduction criteria for apixaban are based on a patient’s age, body weight, and renal function [14]. Because the characteristics of patients with nonvalvular atrial fibrillation using 5 mg BID apixaban and 2.5 BID apixaban may differ [11, 15], clinicians should evaluate clinical outcomes according to apixaban dosage while carefully accounting for these key patient characteristics. Prior studies by Li et al. [16] and Yao et al. [12] included subanalyses using interaction terms to test if the treatment effect on stroke/SE and major bleeding varied between the 2 label-recommended apixaban dose regimens when compared to warfarin using data from United States (US) clinical practice. While Yao et al. found a significant interaction between initial apixaban dose and the treatment effect of apixaban versus warfarin on major bleeding (p = 0.04), a nonsignificant interaction effect was observed for stroke/SE by dose (p = 0.84) [12]. In contrast, the subanalysis by Li et al. found no significant interaction between initial apixaban dose and the treatment effects of apixaban versus warfarin on stroke/SE (p = 0.848) and major bleeding (p = 0.561) [16]. However, comparative effectiveness and safety outcomes for each apixaban dose regimen versus warfarin (in the respective US populations for which the dosages are indicated) have not been available. Therefore, the current study evaluated patient outcomes in the 2 distinct populations for which apixaban was prescribed in US clinical practice, reflecting real-world treatment patterns. In this study, we pooled data from 4 US claims databases to compare baseline characteristics between patients who were prescribed 5 mg BID and 2.5 mg BID apixaban. The risk of stroke/SE and major bleeding associated with 5 and 2.5 mg BID apixaban was also examined and compared to warfarin.

## Methods

A retrospective observational cohort study from January 1, 2012 to September 30, 2015 was conducted using fully anonymized, pooled data from 4 large, nationally representative databases in the US: the Truven MarketScan^®^ Commercial Claims and Encounter and Medicare Supplemental and Coordination of Benefits Database (“MarketScan”), the IMS PharMetrics Plus™ Database (“PharMetrics”), the Optum Clinformatics™ Data Mart (“Optum”), and the Humana Research Database (“Humana”).

The pooled data included patient demographics, enrollment history, and medical and pharmacy claims for more than 163 million members of commercial and Medicare Advantage/supplemental plans. Medical claims from inpatient and outpatient healthcare settings were coded using International Classification of Disease, 9th Revision, Clinical Modification (ICD-9-CM), Current Procedural Terminology, or Healthcare Common Procedure Coding System codes, and pharmacy claims data used the National Drug Code coding system to capture dispensed drugs. Laboratory test results (eg, creatinine clearance) and biomarkers (eg, body weight) are not comprehensively recorded in the 4 claims databases. Further explanation of the data source can be found in a recent publication by Li et al. [16] that details results of a pooled analysis (using data from the same 4 databases outlined above) on the effectiveness and safety of apixaban and warfarin [16]. To date, these databases used in the present study have also been used in previous pooled analyses of various therapeutic areas [16–23].

In each database, patients were identified who met the following criteria: adults (aged ≥18 years) with nonvalvular atrial fibrillation who had a pharmacy claim for apixaban or warfarin during the identification period (January 1, 2013 to September 30, 2015). The first apixaban or warfarin prescription claim date was defined as the index date. Patients were required to have an atrial fibrillation diagnosis (ICD-9-CM code 427.31) before or on the index date and ≥12 months of continuous medical and pharmacy health plan enrollment prior to the index date (baseline period) [24].

Patients were excluded if they had evidence of pregnancy during the study period or valvular heart disease, venous thromboembolism, transient atrial fibrillation (pericarditis, hyperthyroidism, thyrotoxicity), or heart valve replacement/transplant during the 12 months prior to or on the index date. Although the identification period was until September 30, 2015 (a day before the Centers for Medicare & Medicaid Services required implementation of ICD-10 codes [October 1, 2015]), some health plans may have transitioned from ICD-9 to ICD-10 codes earlier. Therefore, patients with any claims using ICD-10 codes during the study period were excluded. Also excluded were patients prescribed any oral anticoagulant within 12 months before the index date or >1 oral anticoagulant on the index date. The index apixaban dose was identified, and patients were categorized as 5 mg BID or 2.5 mg BID apixaban patients.

The primary effectiveness outcome was stroke/SE, and the primary safety outcome was major bleeding; these were identified using the first listed ICD-9-CM diagnosis code of inpatient claims. Stroke/SE events included ischemic stroke, hemorrhagic stroke, and systemic embolism; major bleeding included gastrointestinal bleeding, intracranial hemorrhage, and other major bleeding. The diagnosis codes used for stroke/SE and major bleeding were based on a validated algorithm developed for administrative claims data as well as the criteria for major bleeding as defined by the International Society on Thrombosis and Haemostasis, which were also used in the ARISTOTLE trial (**S1 Table**) [25, 26]. The follow-up period began the day after the index date and continued until censored at the first occurrence of any of the following outcome events: 30 days after the discontinuation date (discontinuation being defined as no evidence of index prescription for 30 days from the last day of supply of the last filled prescription), the switch date to an oral anticoagulant other than that prescribed at index, inpatient death, end of continuous medical and pharmacy enrollment, 1-year after the index date, or the end of the study period (September 30, 2015). Patients were censored 1 year after the index date to balance the follow-up time between the apixaban and warfarin cohorts.

Patient demographics and clinical characteristics during the 12 months prior to and on the index date were measured. Comorbidities and clinical risk scores were assessed using ICD-9-CM codes (eg, renal disease was defined with ICD-9-CM codes for nephritis, nephrotic syndrome, and nephrosis). Stroke and major bleeding risk were assessed using the respective scores of CHA_2_DS_2_-VASc (congestive heart failure, hypertension, aged >75 years, diabetes, prior stroke or transient ischemic attack or thromboembolism plus vascular disease, aged 65–74 years, and sex) and HAS-BLED (hypertension, abnormal renal and liver function, stroke, bleeding, labile international normalized ratios, age >65 years, drugs or alcohol abuse or dependence) [27, 28]. International normalized ratios values were not available in the databases and, therefore, not included in the HAS-BLED score calculation.

To examine differences in outcomes associated with the 2 dosing populations, propensity-score matching (PSM) was conducted between 5 mg BID apixaban and warfarin patients and 2.5 mg BID apixaban and warfarin patients within each database to minimize selection bias and adjust for potential confounders. We performed logistic regressions using the baseline variables of age, sex, US geographic region, Charlson comorbidity index score, baseline bleeding and stroke/SE history, comorbidities, and baseline comedications to obtain the propensity score for the probability of using apixaban. The PSM cohorts were created using the nearest-neighbor-matching algorithm without replacement, with a caliper of 0.01 [29]. The balance of covariates was checked based on standardized differences with a threshold of 10% [30]. After ensuring cohorts were balanced within each of the databases following 1:1 PSM, the resulting patient records were pooled.

A head-to-head comparison between 5 mg BID and 2.5 mg BID apixaban was conducted to compare baseline differences between the 2 cohorts. P-values were calculated from chi-square and t-tests for categorical and continuous variables, respectively.

Incidence rates were calculated as the number of events divided by the time at risk and presented per 100 person-years. The cumulative incidence of major bleeding and stroke was evaluated using Kaplan-Meier survival curves. The risk of stroke/SE and major bleeding between the matched cohorts (5 mg BID apixaban vs warfarin; 2.5 mg BID apixaban vs warfarin) was evaluated using Cox proportional hazards models with robust sandwich estimates [29]. Apixaban (2.5 mg BID or 5 mg BID) or warfarin treatment was included as the independent variable. For the 5 mg BID apixaban analysis, no other covariates were included in the model because the matched cohorts were balanced; however, for the 2.5 mg BID apixaban analysis, age had an imbalance after matching and was, therefore, adjusted in the models. The log-log of the Kaplan-Meier survival curves was visually inspected to check the proportional hazards assumption. A p value <0.05 was considered statistically significant.

A sensitivity analysis was conducted among 2.5 mg BID and 5 mg BID apixaban-matched warfarin patients for the entire follow-up period (not restricted to 1 year).

## Results

**Fig 1:**
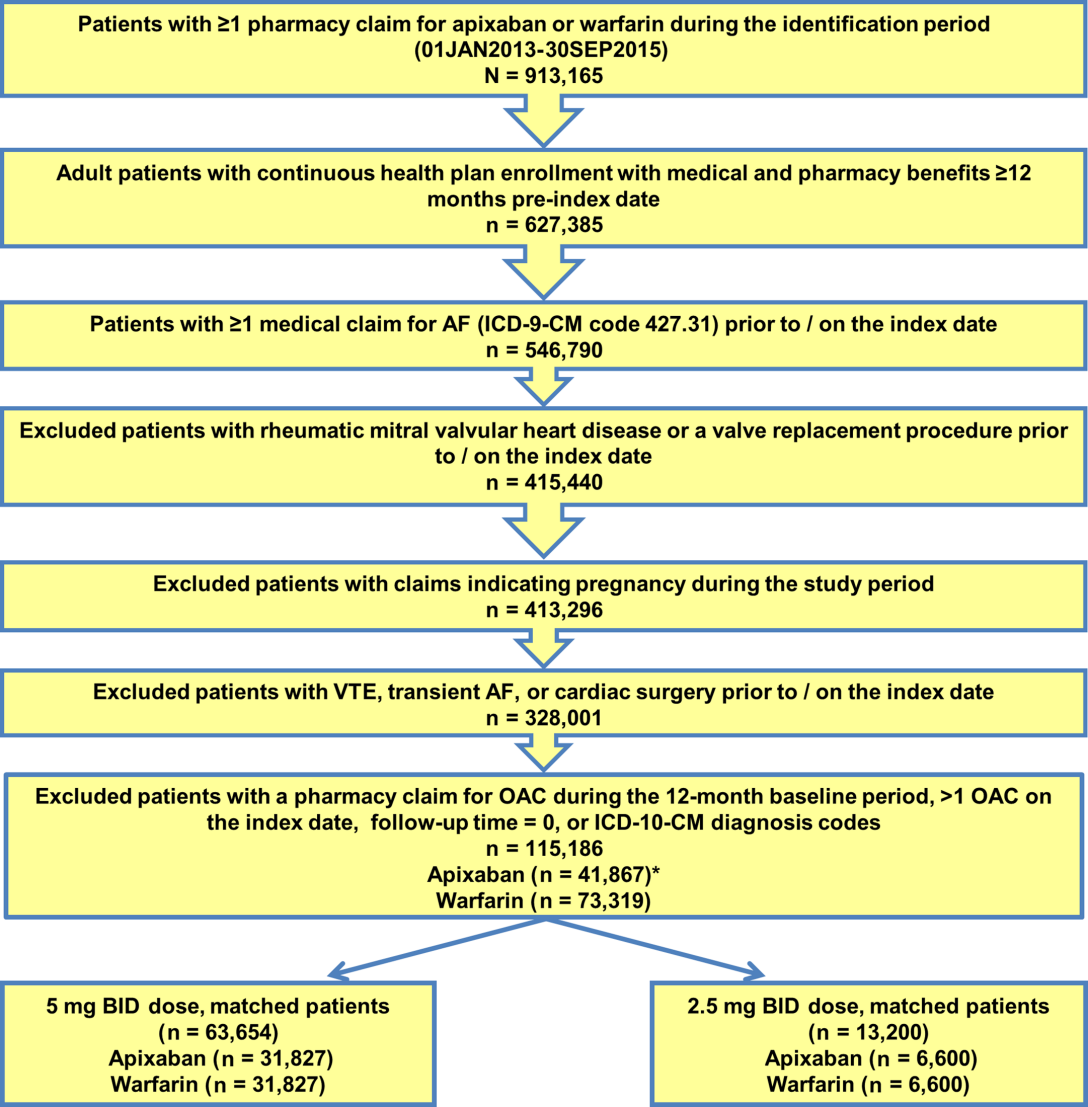
Patient selection flowchart. *15 Patients had both doses of apixaban on the index date, so they were not included in the analysis. AF: atrial fibrillation; BID: twice daily; ICD-10-CM: International Classification of Disease, 10th Revision, Clinical Modification; VTE: venous thromboembolism.

Across all data sources, 115,186 nonvalvular atrial fibrillation patients were identified between January 1, 2013 and September 30, 2015, including 41,867 (36.3%) apixaban and 73,319 (63.7%) warfarin patients (**Fig 1**).

Because 15 apixaban patients had claims for both doses of apixaban on the index date, they were not included in the analysis. Of the 41,852 patients with a single dose of apixaban on the index date, 35,105 (83.9%) were prescribed 5 mg BID and 6747 (16.1%) patients were prescribed 2.5 mg BID. Compared with warfarin patients, 5 mg BID apixaban patients were significantly younger (67.7 years vs 72.6 years, p < 0.001) and had lower stroke and major bleeding risk scores (CHA_2_DS_2_-VASc: 2.9 vs 3.6 and HAS-BLED: 2.4 vs 2.8, p < 0.001). However, 2.5 mg BID apixaban patients were older (82.5 years vs 72.6 years, p < 0.001) and had higher stroke and major bleeding risk scores (CHA_2_DS_2_-VASc: 4.5 vs 3.6 and HAS-BLED: 3.4 vs 2.8, p < 0.001) compared with warfarin patients (**S2 Table**).

After 1:1 PSM, 31,827 5 mg BID apixaban patients were matched to 31,827 warfarin patients, and 6600 2.5 mg BID apixaban patients were matched to 6600 warfarin patients. Baseline characteristics of the matched populations are listed in **Table 1**.

**Table 1:** Baseline characteristics for PSM-adjusted 5 mg BID apixaban and warfarin and 2.5 mg BID apixaban and warfarin patients.

Parameter	5 mg BID Apixaban Cohort		2.5 mg BID Apixaban Cohort			Warfarin Cohort		Warfarin Cohort	
						(5 mg BID Matched)		(2.5 mg BID Matched)	
	N/Mean	%/SD	N/Mean	%/SD	P-value^a^	N/Mean	%/SD	N/Mean	%/SD
**Sample Size**	**31,827**		**6,600**			**31,827**		**6,600**	
**Age, years**	68.6	11.0	82.5	9.5	<0.001	69.2	11.7	80.1	8.5
**18–54**	3058	9.6%	84	1.3%	<0.001	3105	9.8%	82	1.2%
**55–64**	8560	26.9%	288	4.4%	<0.001	8524	26.8%	275	4.2%
**65–74**	10,016	31.5%	602	9.1%	<0.001	9879	31.0%	588	8.9%
**≥75**	10,193	32.0%	5626	85.2%	<0.001	10,319	32.4%	5655	85.7%
**Gender**									
**Male**	20,007	62.9%	2756	41.8%	<0.001	20,048	63.0%	2760	41.8%
**Female**	11,820	37.1%	3844	58.2%	<0.001	11,779	37.0%	3840	58.2%
**United States Geographic Region**									
**Northeast**	4876	15.3%	977	14.8%	0.287	4816	15.1%	983	14.9%
**Midwest**	8657	27.2%	1608	24.4%	<0.001	8614	27.1%	1628	24.7%
**South**	13,428	42.2%	2841	43.0%	0.201	13,405	42.1%	2813	42.6%
**West**	4586	14.4%	1154	17.5%	<0.001	4730	14.9%	1144	17.3%
**Other**	280	0.9%	20	0.3%	<0.001	262	0.8%	32	0.5%
**Baseline Comorbidity**									
**Deyo-Charlson Comorbidity Index Score**	2.3	2.3	3.6	2.7	<0.001	2.3	2.3	3.5	2.7
**CHADS**_**2**_ **score**	1.9	1.2	2.9	1.2	<0.001	1.9	1.2	2.9	1.2
**0**	3491	11.0%	82	1.2%	<0.001	3289	10.3%	61	0.9%
**1**	9522	29.9%	623	9.4%	<0.001	9400	29.5%	604	9.2%
**2**	9991	31.4%	2151	32.6%	0.057	10,384	32.6%	2244	34.0%
**3+**	8823	27.7%	3744	56.7%	<0.001	8754	27.5%	3691	55.9%
**CHA**_**2**_**DS**_**2**_**-VASc score**	3.0	1.7	4.5	1.5	<0.001	3.0	1.7	4.5	1.5
**0**	2381	7.5%	45	0.7%	<0.001	2226	7.0%	27	0.4%
**1**	3781	11.9%	87	1.3%	<0.001	3886	12.2%	91	1.4%
**2**	6555	20.6%	305	4.6%	<0.001	6458	20.3%	306	4.6%
**3**	7388	23.2%	1084	16.4%	<0.001	7566	23.8%	1092	16.5%
**4+**	11,722	36.8%	5079	77.0%	<0.001	11,691	36.7%	5084	77.0%
**3+**	19,110	60.0%	6163	93.4%	<0.001	19,257	60.5%	6,176	93.6%
**HAS-BLED score**^**b**^	2.5	1.3	3.3	1.3	<0.001	2.4	1.3	3.3	1.3
**0**	1858	5.8%	28	0.4%	<0.001	1853	5.8%	23	0.3%
**1**	5734	18.0%	344	5.2%	<0.001	5754	18.1%	357	5.4%
**2**	9487	29.8%	1422	21.5%	<0.001	9638	30.3%	1461	22.1%
**3+**	14,748	46.3%	4806	72.8%	<0.001	14,582	45.8%	4759	72.1%
**Bleeding history**	4922	15.5%	1457	22.1%	<0.001	4780	15.0%	1440	21.8%
**Congestive heart failure**	6835	21.5%	2450	37.1%	<0.001	6804	21.4%	2426	36.8%
**Diabetes mellitus**	10,234	32.2%	2235	33.9%	0.007	10,357	32.5%	2274	34.5%
**Hypertension**	25,907	81.4%	5862	88.8%	<0.001	25,959	81.6%	5880	89.1%
**Renal disease**	5044	15.8%	2535	38.4%	<0.001	5091	16.0%	2546	38.6%
**Liver disease**	1437	4.5%	281	4.3%	0.357	1347	4.2%	260	3.9%
**Myocardial infarction**	2630	8.3%	787	11.9%	<0.001	2532	8.0%	777	11.8%
**Dyspepsia or stomach discomfort**	5219	16.4%	1442	21.8%	<0.001	5089	16.0%	1428	21.6%
**Non-stroke/SE peripheral vascular Disease**	13,686	43.0%	3738	56.6%	<0.001	13,521	42.5%	3788	57.4%
**Stroke/SE**	2835	8.9%	1051	15.9%	<0.001	2775	8.7%	1041	15.8%
**Transient ischemic attack**	1779	5.6%	628	9.5%	<0.001	1736	5.5%	593	9.0%
**Anemia and coagulation defects**	5166	16.2%	1968	29.8%	<0.001	5034	15.8%	1995	30.2%
**Alcoholism**	727	2.3%	72	1.1%	<0.001	718	2.3%	56	0.8%
**Baseline Medication Use**									
**ACE/ARB**	18,558	58.3%	4003	60.7%	<0.001	18,749	58.9%	4047	61.3%
**Amiodarone**	3277	10.3%	961	14.6%	<0.001	3165	9.9%	947	14.3%
**Beta-blockers**	19,050	59.9%	4057	61.5%	0.015	19,000	59.7%	4041	61.2%
**H**_**2**_**-receptor antagonist**	1536	4.8%	454	6.9%	<0.001	1511	4.7%	441	6.7%
**Proton pump inhibitor**	8421	26.5%	2189	33.2%	<0.001	8308	26.1%	2098	31.8%
**Statins**	17,884	56.2%	3961	60.0%	<0.001	18,027	56.6%	3973	60.2%
**Antiplatelets**	4774	15.0%	1424	21.6%	<0.001	4695	14.8%	1431	21.7%
**NSAIDs**	7661	24.1%	1401	21.2%	<0.001	7610	23.9%	1355	20.5%
**Follow-up time (mean, days)**	179.4	163.2	179.1	163.1		199.5	194.8	204.4	192.6
**Median**	119		119			121		129	
**Follow-up time (mean, days) within 1 year**	158.3	114.6	158.2	115.1		164.9	117.5	170.4	117.8
**Median**	119		119			121		129	

ACE: angiotensin-converting enzyme inhibitor; ARB: angiotensin-receptor blocker; CHADS_2_: congestive heart failure, hypertension, age ≥75 years, diabetes mellitus, prior stroke or transient ischemic attack or thromboembolism; CHA_2_DS_2_-VAS_C_: congestive heart failure, hypertension, age ≥75 years, diabetes mellitus, prior stroke or transient ischemic attack or thromboembolism, vascular disease, age 65–74 years, sex category; HAS-BLED: hypertension, abnormal renal and liver function, stroke, bleeding, labile international normalized ratios, elderly, drugs and alcohol; NSAIDs: nonsteroidal anti-inflammatory drugs; PSM: propensity-score–matched; SD: standard deviation; stroke/SE: stroke/systemic embolism.

^a^**Note:** P-values indicate statistical test comparison between 5 mg BID and 2.5 mg BID apixaban patients.

^b^As the international normalized ratio value is not available in the databases, a modified HAS-BLED score was calculated with a range of 0 to 8.

In both the 5 mg BID apixaban and matched warfarin populations, patients had an average age of 69 years, with 37% female. Both cohorts had similar clinical characteristics: mean Charlson comorbidity index, CHA_2_DS_2_-VASc, and HAS-BLED scores were 2.3, 3.0, and 2.5, respectively. During the baseline period, approximately 9% and 15% of patients had a prior stroke/SE and bleed event, respectively. Similarly, 16% of patients had renal disease during the baseline period.

In general, the characteristics of 2.5 mg BID apixaban and matched warfarin patients were well balanced. Age was the only variable that had a standardized difference >10% between the cohorts (2.5 mg BID apixaban: 82.5 years; warfarin: 80.1 years; standardized difference = 26%); all the other characteristics were well balanced (**Table 1**).

The characteristics of patients prescribed 2.5 mg BID apixaban varied substantially from those prescribed 5 mg BID apixaban. Patients prescribed 2.5 mg BID apixaban were significantly older (average age: 82.5 vs 68.6 years, p < 0.001) and more likely to be women (58.2% vs 37.1%, p < 0.001) than those who were prescribed 5 mg BID apixaban. Patients prescribed 2.5 mg BID apixaban had a significantly greater proportion of patients older than 80 years (74.3% vs 16.1%, p < 0.001) compared with those prescribed 5 mg BID apixaban. In addition, more patients aged <75 years (68.0% vs 14.8%, p < 0.001) and <65 years (36.5% vs 5.6%, p < 0.001) were prescribed 5 mg BID than 2.5 mg BID apixaban. Patients prescribed 2.5 mg BID apixaban had a significantly higher proportion of prior stroke/SE during the baseline period compared with those prescribed 5 mg BID apixaban (15.9% vs 8.9%, p < 0.001). In addition, 2.5 mg BID apixaban patients had a significantly higher proportion of prior bleeding compared to 5 mg BID patients (22.0% vs 15.5%, p < 0.001). Also, the prevalence of renal disease was significantly higher among 2.5 mg BID apixaban patients compared with 5 mg BID patients (38.4% vs 15.8%, p < 0.001).

Comorbidity, stroke, and bleeding risk scores for the 2.5 mg BID apixaban patients (ie, mean Charlson comorbidity index, CHA_2_DS_2_-VASc, and HAS-BLED scores) were 3.6, 4.5, and 3.3, respectively; all scores were significantly higher than those for the 5 mg BID apixaban patients (2.3, 3.0, and 2.5, respectively; all p < 0.001). Specifically, a significantly greater proportion of 2.5 mg BID apixaban patients had a CHA_2_DS_2_-VASc score ≥3 (93.4% vs 60.0%, p < 0.001), CHA_2_DS_2_-VASc score ≥4 (77.0% vs 36.8%, p < 0.001), and HAS-BLED score ≥3 (72.8% vs 46.3%, p < 0.001) compared with 5 mg BID apixaban patients.

By restricting the follow-up period to 1 year, the difference in follow-up duration between patients prescribed apixaban and warfarin was reduced. The average follow-up time was approximately 5–6 months in the matched populations. Although most patients (85%) had a follow-up shorter than 1 year, the maximum follow-up was 2.7 years.

When compared with matched warfarin patients, 5 mg BID apixaban patients had a lower incidence of stroke/SE (2.2 vs 3.0 per 100 person-years; **Table 2**).

**Table 2:** Number of events and incidence rates of clinical outcomes within 1 year for 5 mg BID apixaban and warfarin patients.

	Warfarin Cohort		5 mg BID Apixaban Cohort	
	n = 31,827		n = 31,827	
	Patients With Event	Incidence Rate^a^	Patients With Event	Incidence Rate^a^
**Stroke/SE**	440	3.04	299	2.15
**Ischemic stroke**	368	2.54	251	1.80
**Hemorrhagic stroke**	63	0.43	47	0.34
**SE**	29	0.20	11	0.08
**Major bleeding**	977	6.80	563	4.05
**Intracranial hemorrhage**	142	0.98	86	0.61
**Gastrointestinal bleeding**	476	3.29	288	2.07
**Other major bleeding**	436	3.01	232	1.66

^a^Event rates are shown per 100 person-years.

BID: twice daily; SE: systemic embolism.

**Fig 2:**
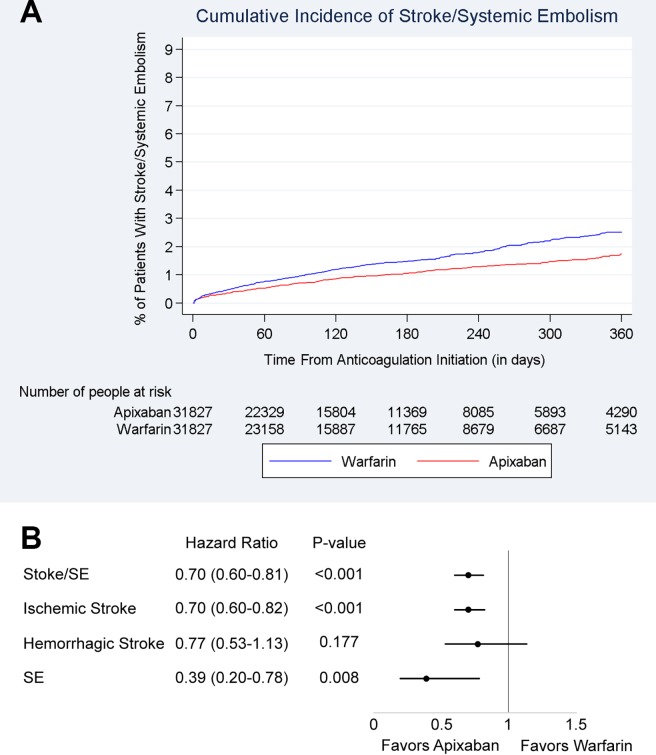
Cumulative incidence and hazard ratios of stroke/systemic embolism among 5 mg BID apixaban/warfarin patients. **(A)** Cumulative incidence of stroke/SE among propensity-score–matched 5 mg BID apixaban and warfarin patients. **(B)** Hazard ratios of stroke/SE for propensity-score–matched 5 mg BID apixaban and warfarin patients. BID, twice daily; stroke/SE, stroke/systemic embolism.

The cumulative incidence of stroke/SE is shown in **Fig 2A**.

The 5 mg BID apixaban was associated with a 30% lower risk of stroke/SE (hazard ratio [HR]: 0.70, 95% confidence interval [CI]: 0.60–0.81) within 1 year of treatment initiation compared with warfarin. Patients prescribed 5 mg BID apixaban also had a 30% reduction in ischemic stroke (HR: 0.70, 95% CI: 0.60–0.82) and a 61% reduction in SE (HR: 0.39, 95% CI: 0.20–0.78). Apixaban patients had a nonsignificant trend toward a lower risk of hemorrhagic stroke (HR: 0.77, 95% CI: 0.53–1.13) compared with warfarin patients (**Fig 2B**).

The incidence of stroke/SE was 3.5 and 5.3 per 100 person-years among the 2.5 mg BID apixaban and matched warfarin patients, respectively (**Table 3**).

**Table 3:** Number of events and incidence rates of clinical outcomes within 1 year for 2.5 mg BID apixaban and warfarin patients.

	Warfarin Cohort n = 6600		2.5 mg BID Apixaban Cohort	
			n = 6600	
	Patients With Event	Incidence Rate^a^	Patients With Event	Incidence Rate^a^
**Stroke/SE**	163	5.28	101	3.51
**Ischemic stroke**	136	4.40	82	2.84
**Hemorrhagic stroke**	23	0.74	13	0.45
**SE**	11	0.35	6	0.21
**Major bleeding**	326	10.64	188	6.56
**Intracranial hemorrhage**	54	1.73	29	1.00
**Gastrointestinal bleeding**	159	5.14	89	3.09
**Other bleeding**	136	4.39	86	2.98

^a^Event rates are shown per 100 person-years.

BID: twice daily; SE: systemic embolism.

**Fig 3:**
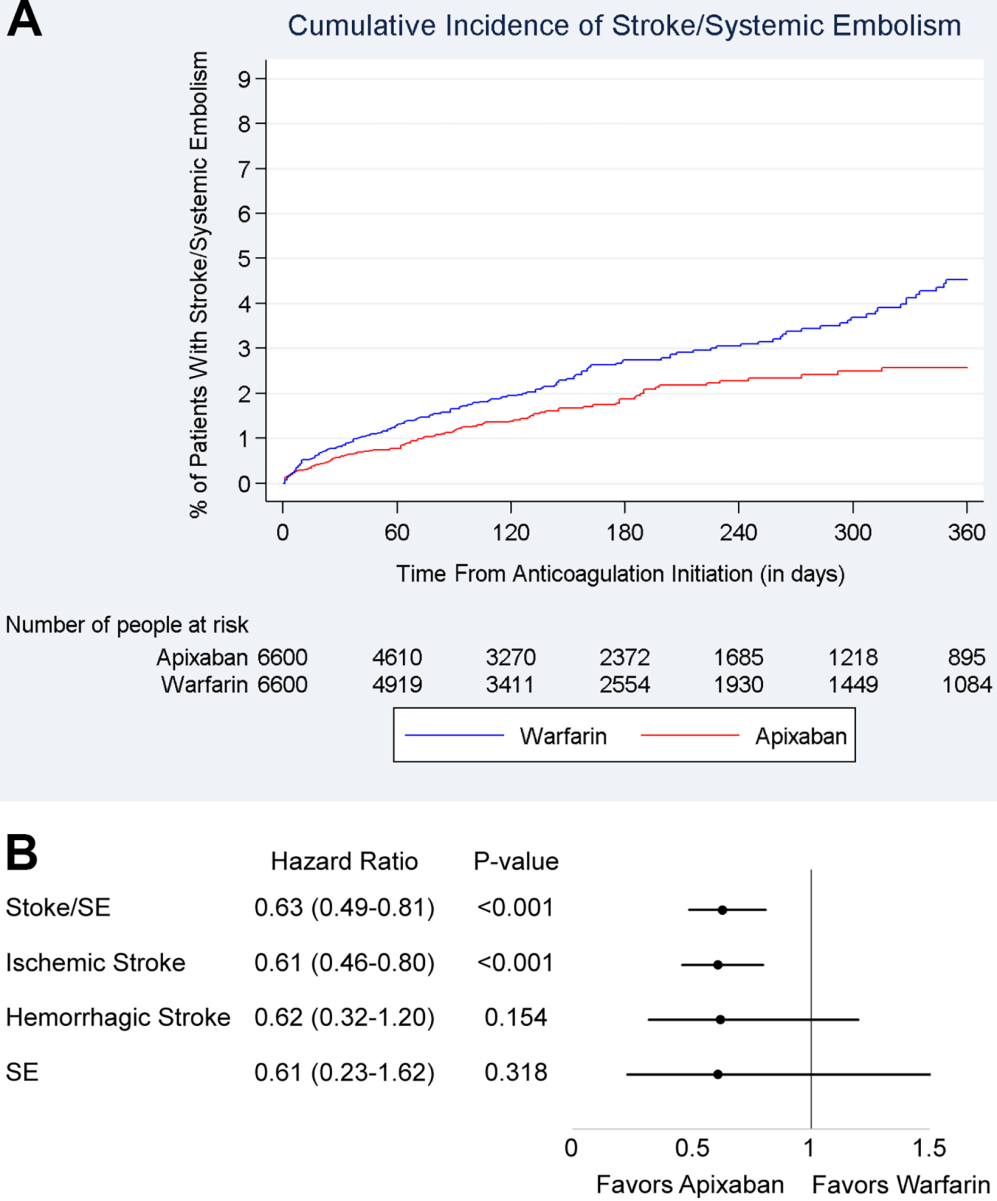
Cumulative incidence and hazard ratios of stroke/systemic embolism among 2.5 mg BID apixaban/warfarin patients. **(A)** Cumulative incidence of stroke/SE among propensity-score–matched 2.5 mg BID apixaban and warfarin patients. **(B)** Hazard ratios of stroke/SE for propensity-score–matched 2.5 mg BID apixaban and warfarin patients. Footnote: BID: twice daily; stroke/SE, stroke/systemic embolism.

The cumulative incidence of stroke/SE is shown in **Fig 3A**.

Compared with warfarin, 2.5 mg BID apixaban was associated with a 37% lower risk of stroke/SE (HR: 0.63, 95% CI: 0.49–0.81), driven by a 39% reduction in ischemic stroke (HR: 0.61, 95% CI: 0.46–0.80). Apixaban patients had a nonsignificant trend toward a lower risk of hemorrhagic stroke (HR: 0.62, 95% CI: 0.32–1.20) and SE (HR: 0.61, 95% CI: 0.23–1.62) compared with warfarin (**Fig 3B**).

**Fig 4:**
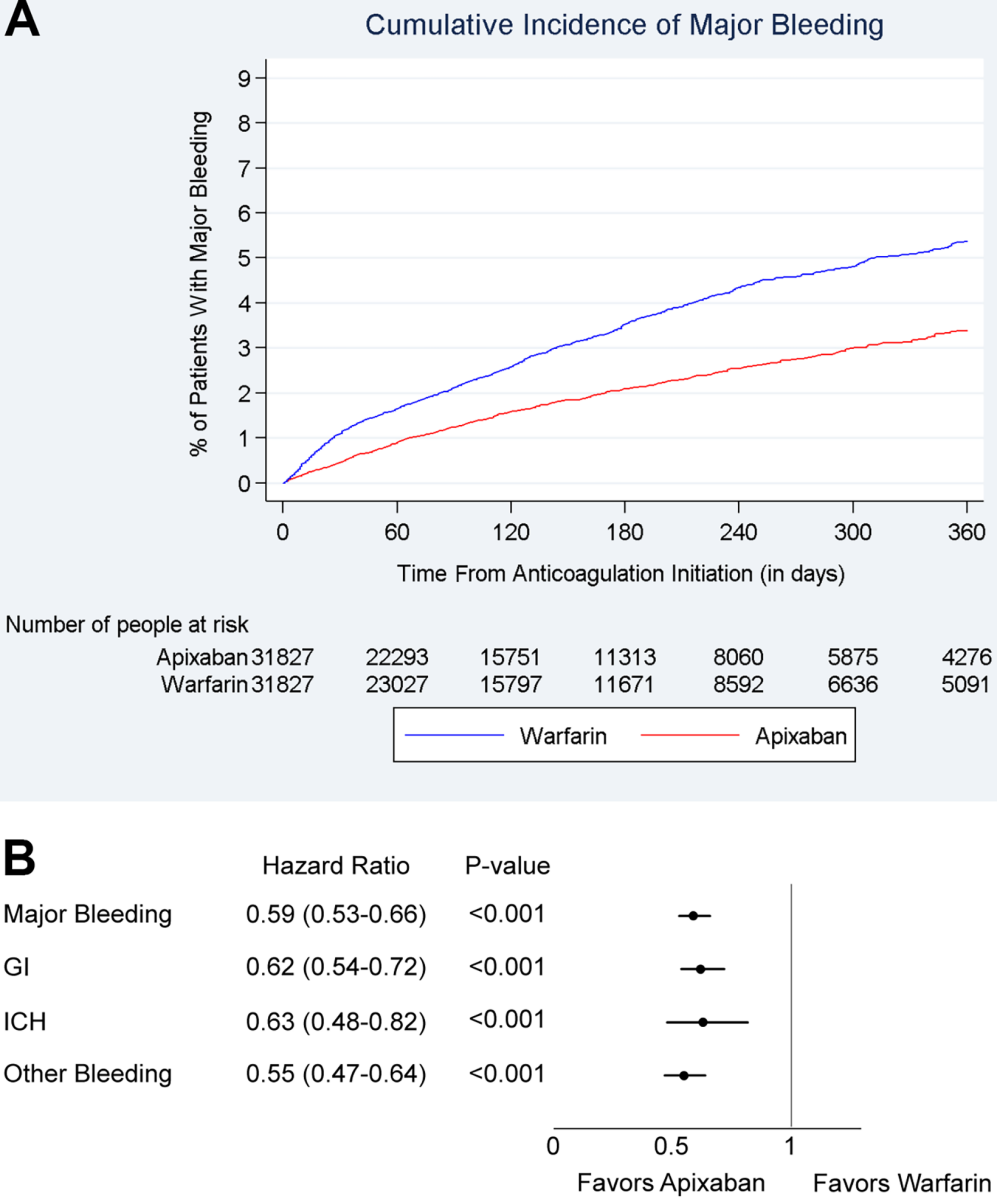
Cumulative incidence and hazard ratios of major bleeding among 5 mg BID apixaban and warfarin patients. **(A)** Cumulative incidence of major bleeding among propensity-score–matched 5 mg BID apixaban and warfarin patients. **(B)** Hazard ratio of major bleeding for propensity-score–matched 5 mg BID apixaban and warfarin patients. BID, twice daily.

The incidence rate of major bleeding was lower among 5 mg BID apixaban patients compared with matched warfarin patients (4.1 vs 6.8 per 100 person-years). The cumulative incidence of major bleeding is shown in **Fig 4A**.

Patients prescribed 5 mg BID apixaban had a 41% lower risk of major bleeding (HR: 0.59, 95% CI: 0.53–0.66) within 1 year of treatment initiation compared with those prescribed warfarin. This reduced major bleeding risk was driven by a reduced risk of gastrointestinal bleeding (HR: 0.62, 95% CI: 0.54–0.72), intracranial hemorrhage (HR: 0.63, 95% CI: 0.48–0.82), and other major bleeding (HR: 0.55, 95% CI: 0.47–0.64) (**Fig 4B**).

**Fig 5:**
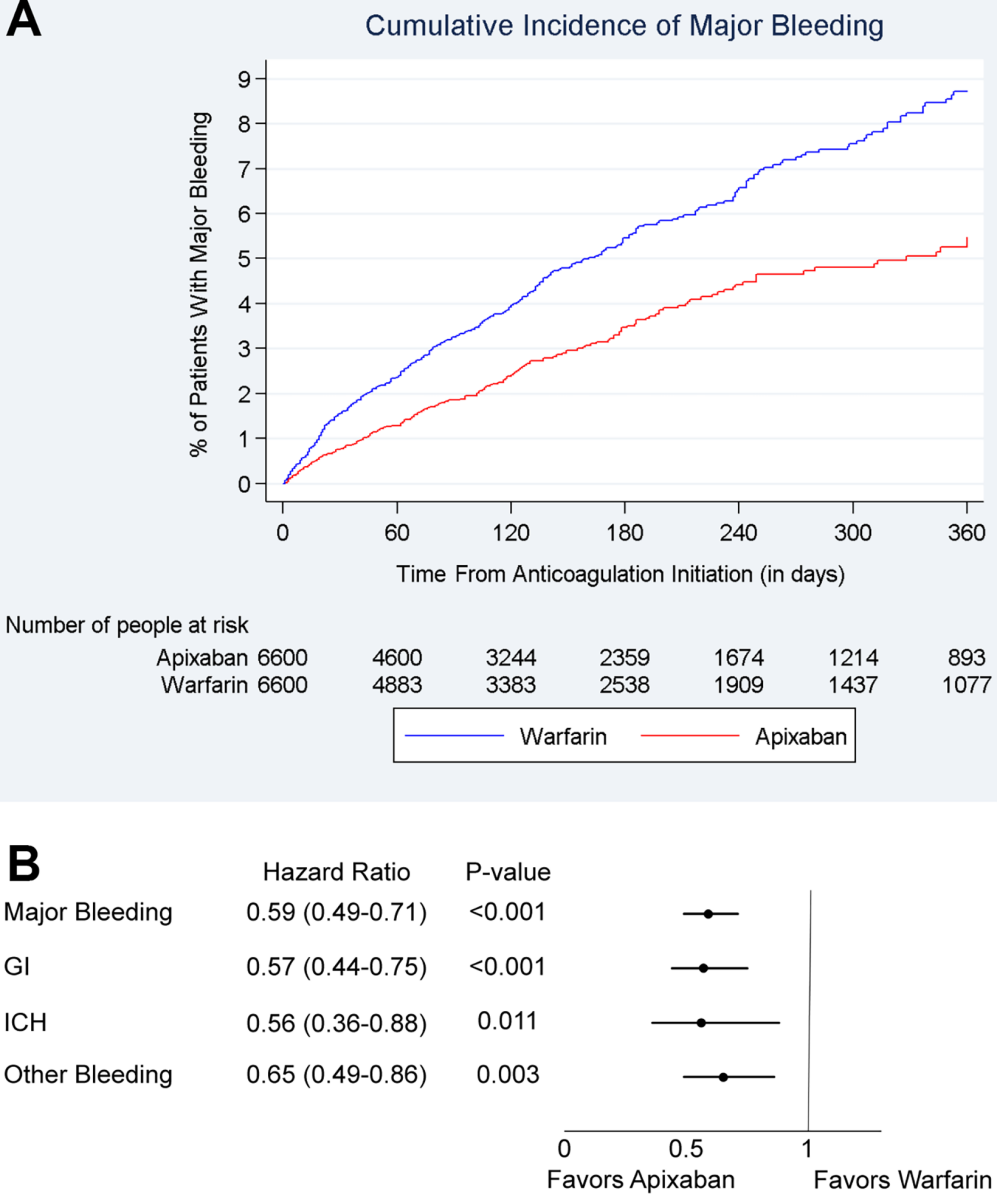
Cumulative incidence and hazard ratios of major bleeding among 2.5 mg BID apixaban and warfarin patients. **(A)** Cumulative incidence of major bleeding among propensity-score–matched 2.5 mg BID apixaban and warfarin patients**. (B)** Hazard ratio of major bleeding for propensity-score–matched 2.5 mg BID apixaban and warfarin patients. BID: twice daily; GI: gastrointestinal bleeding; ICH: intracranial hemorrhage.

The incidence of major bleeding was 6.6 and 10.6 per 100 person-years for 2.5 mg BID apixaban and matched warfarin patients, respectively (**Table 3**). The cumulative incidence of major bleeding is shown in **Fig 5A**.

Patients prescribed 2.5 mg BID apixaban were associated with a 41% lower risk of major bleeding (HR: 0.59, 95% CI: 0.49–0.71) within 1 year of treatment initiation, a 43% reduction in gastrointestinal bleeding (HR: 0.57, 95% CI: 0.44–0.75), 44% reduction in intracranial hemorrhage (HR: 0.56, 95% CI: 0.36–0.88), and 35% reduction in other major bleeding (HR: 0.65, 95% CI: 0.49–0.86) compared with those prescribed warfarin (**Fig 5B**).

In a sensitivity analysis, the risk of major bleeding and stroke over the entire follow-up period was examined for both matched populations. Patients prescribed 5 mg BID apixaban had a significantly lower risk of stroke (HR: 0.70, 95% CI: 0.60–0.80) and major bleeding (HR: 0.59, 95% CI: 0.54–0.65) compared with warfarin. Patients prescribed 2.5 mg BID apixaban also had a significantly lower risk of stroke (HR: 0.64, 95% CI: 0.50–0.82) and major bleeding (HR: 0.63, 95% CI: 0.53–0.74) compared with those prescribed warfarin.

## Discussion

In this real-world retrospective observational analysis, our principal findings are as follows: (I) patients prescribed 5 mg apixaban BID had different characteristics from those prescribed 2.5 mg BID apixaban, and (II) 5 mg BID and 2.5 mg BID apixaban were associated with significantly lower risk of stroke/SE and major bleeding compared with warfarin when assessed as 2 distinct patient populations. To our knowledge, this is the first dose-specific analysis comparing 5 mg BID and 2.5 mg BID apixaban with warfarin using US claims data.

In the current study, baseline characteristics were compared between patients who took 5 mg BID apixaban and those who took 2.5 mg BID apixaban. Apixaban dosages were associated with markedly different patient characteristics. Patients prescribed apixaban 2.5 mg BID were substantially older, mostly women, had clinically more severe comorbidities, and were more likely to have a history of stroke and bleeding compared with those prescribed 5 mg BID apixaban. In addition, 2.5 mg BID apixaban patients were more than twice as likely to have renal disease compared with 5 mg BID apixaban patients. In this real-world study, 5 mg BID and 2.5 mg BID apixaban were observed to be used in the patient subgroups that differed widely in age and clinical characteristics. Although it cannot be ascertained from claims data whether dose selection matched the label-indicated criteria for 2.5 mg BID use, higher mean age and more prevalent renal disease in the 2.5 mg BID apixaban group were consistent with the criteria.

In the current study, 5 mg BID and 2.5 mg BID apixaban patients were associated with lower risks of stroke/SE and major bleeding compared with matched warfarin patients. These reductions were consistent for 5 mg BID and 2.5 mg BID apixaban compared with warfarin. Patients prescribed 5 mg BID apixaban had a 30% reduction in risk of stroke/SE compared with warfarin, and those prescribed 2.5 mg BID apixaban had a 37% reduction in risk for stroke/SE. In addition, the safety analysis showed that 5 mg BID apixaban versus warfarin, and 2.5 mg BID apixaban versus warfarin, had the same magnitude of risk reduction (41%) for major bleeding. In the sensitivity analysis, the results were consistent when the entire follow-up period was used.

In the ARISTOTLE trial, apixaban use showed a 21% lower risk of stroke/SE and a 31% lower risk of major bleeding compared with warfarin use [5]. Although the ARISTOTLE trial did not evaluate stroke and major bleeding in 5 mg BID and 2.5 mg BID apixaban doses separately due to the small sample size of the latter group, previous ARISTOTLE trial subgroup analysis did not find a significant interaction between dose and treatment effect when evaluating stroke/SE and major bleeding [5].

In previously published US real-world studies, most apixaban dose-related analyses were treatment-by-dose interaction or sensitivity analysis with a 5 mg BID apixaban regimen restriction. In a recent publication using OptumLabs data, apixaban patients (n = 7695) were shown to have a significantly lower risk of stroke/SE and major bleeding compared with warfarin patients [12]. Subgroup analyses based on apixaban dose regimens indicated no significant treatment-by-dose interaction for stroke/SE (p = 0.84), but significant interaction for major bleeding (p = 0.04) [12]. In the OptumLabs study, 5 mg BID apixaban was associated with a 62% lower risk of major bleeding (HR: 0.38, 95% CI: 0.28–0.53) compared with warfarin. The HR for major bleeding risk between 2.5 mg BID apixaban and warfarin was <1, but not statistically significant (HR: 0.74, 95% CI: 0.44–1.25), which may have been due to the small sample size of the 2.5 mg BID apixaban group (n < 1400) [12]. Another recent publication based on a pooled analysis of 4 US claims databases also found lower risk of stroke/SE and major bleeding in apixaban patients (n = 38,470, including 6568 on 2.5 mg BID) compared with warfarin patients. However, the subgroup interaction analysis on dose regimen did not result in statistically significant differences in the respective treatment effect between the 2 dose regimens [16]. In a sensitivity analysis of a recent comparative safety study using MarketScan data, 5 mg BID apixaban patients (n = 5961) had a 45% lower risk of major bleeding compared with warfarin patients (HR: 0.55; 95% CI: 0.39–0.77) [9].

Another recently published study using OptumLabs data [13] directly compared outcomes between patients treated with 5 mg BID apixaban and those treated with 2.5 mg BID apixaban, without including warfarin patients as a comparator group. Although data on patient body weight were not available and laboratory values on renal function were available only for approximately one-third of patients in this analysis, the findings suggest that NOAC dosing in real-world practice may be inconsistent with recommendations in drug labeling. In addition, dosing that is inconsistent with recommendations in labeling may impact outcomes. Among apixaban patients without a serum creatinine level ≥ 1.5 mg/dL (ie, without a renal indication for dose reduction), authors reported elevated risk of stroke/SE for those on 2.5 mg BID apixaban (n = 550) compared with those on 5 mg BID apixaban (n = 550; the 2 groups were matched with propensity score). This finding suggests the importance of appropriate dosing of apixaban according to its label. A limitation of the analysis was the relatively small sample size and corresponding low number of events (eg, the comparison described above was based on only 7 stroke/SE events).

The use of 2.5 mg BID apixaban is more prevalent in European countries (30% to 40%) compared with the United States (10% to 20%) [9, 11, 12, 15, 31–35]. Using the Danish National Patient Register databases, 2 recently published studies separately compared the effectiveness and safety of 5 mg BID apixaban (n = 6349) and 2.5 mg BID apixaban (n = 4400) with warfarin in Danish clinical practice [11, 15]. Inverse probability of treatment-weighted methods were used to control for differences in patient characteristics, but residual confounding may still exist as mentioned by the authors. An intent-to-treat approach was applied for all endpoints in both studies without censoring the follow-up time when a patient discontinued index therapy or switched to a different therapy. The study comparing 2.5 mg BID apixaban with warfarin showed that patients prescribed 2.5 mg BID apixaban were older and had higher stroke and bleeding risk as measured by CHA_2_DS_2_-VASc and HAS-BLED scores [11]. In a study comparing 5 mg BID apixaban with warfarin, 5 mg BID apixaban was found to be associated with a lower risk of major bleeding but no significant difference in the risk of ischemic stroke/SE [15]. The analysis comparing 2.5 mg BID apixaban with warfarin did not find significant differences in the risk of ischemic stroke/SE and major bleeding between the 2 cohorts [11].

These findings from the Danish registries are somewhat different from our current study. The different findings may be related to the variance in patient population and prescription pattern (eg, the ratio of 5 mg BID and 2.5 mg BID apixaban patients was approximately 1.5:1 in Danish studies but approximately 5:1 in the current study), sample size (10,749 apixaban patients in Danish studies and 38,427 apixaban patients in the current study), endpoint selection (eg, ischemic stroke/SE vs stroke/SE as the primary effectiveness measure), and statistical methods (eg, inverse probability of treatment-weighted method vs PSM method, and whether censoring follow-up when discontinuation or switch occurred) [11, 15].

The similar effectiveness and safety outcomes being observed for the 5 mg BID and 2.5 mg BID of apixaban in this study do not indicate that the dose regimens should be considered therapeutically equivalent or interchangeable. Use of 2.5 mg BID apixaban should follow therapeutic labels. The unique patient characteristics associated with the 2.5 mg BID selection (older age and higher prevalence of renal disease) are likely important factors that helped achieve the lower risk of stroke/SE and major bleeding for 2.5 mg BID apixaban patients compared with warfarin in this study. These factors are generally consistent with prescribing information recommendations.

A key strength of this study is the size of the sample of patients we were able to obtain by pooling matched populations from 4 large, nationally representative US claims databases. By combining data sources, we were able to obtain a much larger sample size of apixaban patients than previously published studies, increasing the statistical power and allowing us to evaluate effectiveness and safety outcomes not only among patients with commonly used 5 mg BID apixaban but also among less prevalent 2.5 mg BID apixaban patients. The pooling of 4 data sources also improves the generalizability of our study findings. Furthermore, our study is the first analysis in which PSM was completed separately between each apixaban dose and warfarin.

Our study has several limitations. First, due to the retrospective observational design, results are estimates of statistical association, and no causal relationships should be inferred. PSM was applied to the cohorts to reduce confounding; however, residual confounding from unmeasured variables, such as over-the-counter use of aspirin or changes in warfarin dose, may remain. Laboratory data such as creatinine clearance levels or international normalized ratios were not comprehensively captured in the administrative claims data. We were not able to ascertain whether the dose selection matches the indicated criteria because information regarding body weight and serum creatinine was not available; however, the increased age and higher percentage of renal disease in the 2.5 mg BID apixaban group are consistent with the indicated criteria. Also, misclassification errors may have occurred because some ICD-9-CM codes may have been incorrectly recorded, misused, or never entered. This study included only treatment-naïve apixaban and warfarin patients to avoid potential confounding associated with therapy switch. Patients who switched from warfarin to apixaban may have done so due to poor quality of international normalized ratio control, which cannot be measured in the data source, or may be different from patients who continued using warfarin in other ways that could introduce bias. Although the sample size used for this observational study is considerably larger than that of the 2.5 mg dosing group included in the ARISTOTLE trial (6,600 patients vs 428 patients, respectively), study results should be considered for hypothesis generation only and not be considered as conclusive [5].

Several limitations related to the data source were present in this analysis. Although overlapping at the health plan level is expected to be minimal across different health plans contributing data to any of the 4 databases, duplicate patient records may exist across the databases, especially between the 2 employer-based claims databases (MarketScan and PharMetrics); however, the percentage of those potential duplicates in a previously published pooled analysis of the 2 databases was estimated to be small (0.5%) and, therefore, not likely to affect the results [17]. There is also the potential for observed and unobserved heterogeneity among the 4 databases due to differences in health plans and patient populations covered in each database. To address this, PSM was conducted within each database prior to pooling the matched patient records across databases. In an exploratory analysis, the interaction terms between treatment effect and each database were not significant and the results were consistent across the 4 databases. Lastly, the data source did not comprehensively contain renal function laboratory values or body weight, and therefore it could not be fully ascertained whether patients met age, body weight, and creatinine level criteria for dose reduction, as defined in the ARISTOTLE trial and the prescribing information for apixaban [5]. Although some US data sources (particularly those linking or integrating data from both insurance claims and electronic medical records) may have comprehensive body weight and renal function laboratory values available, feasibility assessment and previous literature suggested that the sample size of 2.5 mg BID apixaban patients in those data sources would be quite small. For example, only 550 2.5 BID apixaban patients were included in a recently published propensity-score matched analysis using OptumLabs data [13]. The current study was conducted in an attempt to better understand characteristics and outcomes associated with 2.5 mg BID apixaban patients based on a large sample of those patients treated in US clinical practice.

Finally, this real-world study differs from clinical trials in several ways [36]. First, the identification of stroke/SE and major bleeding events was based on administrative claims and was not verified with a review of medical records; in contrast, these events were adjudicated in the ARISTOTLE trial [5]. Inclusion and exclusion criteria for the present real-world study were less stringent than those required in the clinical trial, and the patient sample size was larger. In addition, patients receiving warfarin in routine clinical practice may have had a reduced time in therapeutic range (TTR) compared to those in a clinical trial—potentially due to less frequent international normalized ratio (INR) monitoring and warfarin management—which may have led to higher rates of ischemic stroke and gastrointestinal bleeding observed in the present study versus those in the clinical trial setting [37]. Although INR values for patients receiving treatment with warfarin were not available in the databases used in this study, prior research using ARISTOTLE trial data has suggested that the treatment effects of apixaban compared with warfarin on stroke/SE and major bleeding appear similar across the range of predicted quality of INR control [38].

## Conclusion

In this real-world study, 5 mg BID and 2.5 mg BID doses of apixaban were assessed for 2 patient groups that differed widely in age and clinical characteristics. Each apixaban dose was associated with a lower risk of stroke/SE and major bleeding compared with warfarin in the distinct population for which it is currently prescribed in US clinical practice. This study provides observational evidence to supplement the results of the ARISTOTLE trial, which found no significant interaction between dose and treatment effect regarding stroke/SE and major bleeding [5]. To confirm the findings of this observational study, additional research should be performed using different data sources (ideally data sources with comprehensive body weight and renal function laboratory values) and larger patient sample sizes.

## Supporting information

S1 Table ICD-9-CM codes for stroke/systemic embolism and major bleeding endpoints. (DOCX)

S2 Table Baseline characteristics for 5 mg BID apixaban, 2.5 mg BID apixaban, and warfarin patients (prior to matching). (DOCX)
