# Supplementary material for: Apixaban 5 and 2.5 mg twice-daily versus warfarin for stroke prevention in nonvalvular atrial fibrillation patients: Comparative effectiveness and safety evaluated using a propensity-score-matched approach
Source: PLoS One. 2018 Jan 26;13(1):e0191722. doi: 10.1371/journal.pone.0191722 (PMC5786316; doi:10.1371/journal.pone.0191722)
Supplement: S1 Table — (DOCX) [file pone.0191722.s001.docx]

**Supplemental Table 1. ICD-9-CM Codes for Stroke/Systemic Embolism and Major Bleeding Endpoints**

| Diagnosis | ICD-9 Diagnosis and Procedure Codes |
| --- | --- |
| Hemorrhagic stroke | 430.xx-432.xx  Cases were excluded if traumatic brain injury (ICD-9: 800-804, 850-854) was present during hospitalization |
| Ischemic stroke | 433.x1, 434.x1, 436 |
| Systemic embolism | 444.x, 445.x |
| Major gastrointestinal bleeding event | 456.0, 456.20, 530.82, 531.0x, 531.2x, 531.4x, 531.6x, 532.0x, 532.2x, 532.4x, 532.6x, 533.0x, 533.2x, 533.4x, 533.6x, 534.0x, 534.2x, 534.4x, 534.6x, 535.01, 535.11, 535.21, 535.31, 535.41, 535.51, 535.61, 537.83, 562.02, 562.03, 562.12, 562.13, 568.81, 569.3, 569.85, 578.x  Procedure Code: 44.43 |
| Major intracranial hemorrhage | 430, 431, 432.0, 432.1, 432.9, 852.0x, 852.2x, 852.4x, 853.0x |
| Major other hemorrhage | 285.1, 360.43, 362.43, 362.81, 363.61, 363.62, 363.72, 364.41, 372.72, 374.81, 376.32, 377.42, 379.23, 423.0x, 596.7x, 599.7x 602.1x, 620.1, 621.4, 626.2, 626.5, 626.7, 626.8, 626.9, 719.1x, 782.7, 784.7, 784.8, 786.3x, 958.2, 997.02, 998.11  Procedure codes: 99.04 |

ICD-9-CM: International Classification of Disease, 9th Revision, Clinical Modification.
