# Supplementary material for: Apixaban 5 and 2.5 mg twice-daily versus warfarin for stroke prevention in nonvalvular atrial fibrillation patients: Comparative effectiveness and safety evaluated using a propensity-score-matched approach
Source: PLoS One. 2018 Jan 26;13(1):e0191722. doi: 10.1371/journal.pone.0191722 (PMC5786316; doi:10.1371/journal.pone.0191722)
Supplement: S2 Table — (DOCX) [file pone.0191722.s002.docx]

**Supplemental Table 2. Baseline Characteristics for 5 mg BID Apixaban, 2.5 mg BID Apixaban, and Warfarin Patients (Prior to Matching)**

| Parameter | Warfarin Cohort (Reference) | | 5 mg BID Apixaban | | | 2.5 mg BID Apixaban | | |
| --- | --- | --- | --- | --- | --- | --- | --- | --- |
|  | N/Mean | %/SD | N/Mean | %/SD | P-value* | N/Mean | %/SD | P-value |
| Sample size | 73,319 | | 35,105 | |  | 6747 | |  |
| Age, years | 72.6 | 11.4 | 67.7 | 11.2 | <0.001 | 82.5 | 9.5 | <0.001 |
| 18–54 | 4460 | 6.1% | 4101 | 11.7% | <0.001 | 84 | 1.2% | <0.001 |
| 55–64 | 14,101 | 19.2% | 10,042 | 28.6% | <0.001 | 288 | 4.3% | <0.001 |
| 65–74 | 20,280 | 27.7% | 10,716 | 30.5% | <0.001 | 602 | 8.9% | <0.001 |
| ≥75 | 34,478 | 47.0% | 10,246 | 29.2% | <0.001 | 5773 | 85.6% | <0.001 |
| Gender | | | | | | | | |
| Male | 43,697 | 59.6% | 22,343 | 63.7% | <0.001 | 2776 | 41.1% | <0.001 |
| Female | 29,622 | 40.4% | 12,762 | 36.4% | <0.001 | 3971 | 58.9% | <0.001 |
| US Geographic Region | | | | | | | | |
| Northeast | 11,649 | 15.9% | 5102 | 14.5% | <0.001 | 980 | 14.5% | 0.003 |
| Midwest | 22,958 | 31.3% | 8838 | 25.2% | <0.001 | 1610 | 23.9% | <0.001 |
| South | 23,288 | 31.8% | 16,223 | 46.2% | <0.001 | 2979 | 44.2% | <0.001 |
| West | 14,877 | 20.3% | 4613 | 13.1% | <0.001 | 1158 | 17.2% | <0.001 |
| Other | 547 | 0.8% | 329 | 0.9% | 0.001 | 20 | 0.3% | <0.001 |
| Baseline Comorbidity |  |  |  |  |  |  |  |  |
| Deyo-Charlson comorbidity index | 3.0 | 2.7 | 2.2 | 2.3 | <0.001 | 3.7 | 2.7 | <0.001 |
| CHADS_2_ score | 2.3 | 1.3 | 1.8 | 1.2 | <0.001 | 2.9 | 1.2 | <0.001 |
| 0 | 5114 | 7.0% | 4008 | 11.4% | <0.001 | 82 | 1.2% | <0.001 |
| 1 | 15,714 | 21.4% | 11,282 | 32.1% | <0.001 | 623 | 9.2% | <0.001 |
| 2 | 23,219 | 31.7% | 10,763 | 30.7% | <0.001 | 2186 | 32.4% | 0.217 |
| 3+ | 29,272 | 39.9% | 9052 | 25.8% | <0.001 | 3856 | 57.2% | <0.001 |
| CHADS_2_-VASc score | 3.6 | 1.7 | 2.9 | 1.7 | <0.001 | 4.5 | 1.5 | <0.001 |
| 0 | 3188 | 4.4% | 2815 | 8.0% | <0.001 | 45 | 0.7% | <0.001 |
| 1 | 5525 | 7.5% | 4778 | 13.6% | <0.001 | 87 | 1.3% | <0.001 |
| 2 | 11,100 | 15.1% | 7636 | 21.8% | <0.001 | 305 | 4.5% | <0.001 |
| 3 | 16,208 | 22.1% | 7923 | 22.6% | 0.086 | 1088 | 16.1% | <0.001 |
| 4+ | 37,298 | 50.9% | 11,953 | 34.1% | <0.001 | 5222 | 77.4% | <0.001 |
| 3+ | 53,506 | 73.0% | 19,876 | 56.6% | <0.001 | 6310 | 93.5% | <0.001 |
| HAS-BLED score | 2.8 | 1.4 | 2.4 | 1.3 | <0.001 | 3.4 | 1.3 | <0.001 |
| 0 | 2661 | 3.6% | 2119 | 6.0% | <0.001 | 28 | 0.4% | <0.001 |
| 1 | 9536 | 13.0% | 6805 | 19.4% | <0.001 | 345 | 5.1% | <0.001 |
| 2 | 19,569 | 26.7% | 10,641 | 30.3% | <0.001 | 1426 | 21.1% | <0.001 |
| 3+ | 41,553 | 56.7% | 15,540 | 44.3% | <0.001 | 4948 | 73.3% | <0.001 |
| Bleeding history | 15,257 | 20.8% | 5176 | 14.7% | <0.001 | 1492 | 22.1% | 0.012 |
| CHF | 22,143 | 30.2% | 7075 | 20.2% | <0.001 | 2543 | 37.7% | <0.001 |
| Diabetes mellitus | 27,465 | 37.5% | 10,933 | 31.1% | <0.001 | 2273 | 33.7% | <0.001 |
| Hypertension | 60,946 | 83.1% | 28,578 | 81.4% | <0.001 | 6006 | 89.0% | <0.001 |
| Renal disease | 18,781 | 25.6% | 5133 | 14.6% | <0.001 | 2658 | 39.4% | <0.001 |
| Liver disease | 3557 | 4.9% | 1612 | 4.6% | 0.061 | 289 | 4.3% | 0.037 |
| Myocardial infarction | 8049 | 11.0% | 2753 | 7.8% | <0.001 | 823 | 12.2% | 0.002 |
| Dyspepsia or stomach discomfort | 13,505 | 18.4% | 5753 | 16.4% | <0.001 | 1492 | 22.1% | <0.001 |
| Non-stroke/SE peripheral vascular disease | 35,679 | 48.7% | 14,640 | 41.7% | <0.001 | 3849 | 57.1% | <0.001 |
| Stroke/SE | 9734 | 13.3% | 2943 | 8.4% | <0.001 | 1074 | 15.9% | <0.001 |
| TIA | 4990 | 6.8% | 1905 | 5.4% | <0.001 | 660 | 9.8% | <0.001 |
| Anemia and coagulation defects | 18,772 | 25.6% | 5240 | 14.9% | <0.001 | 2031 | 30.1% | <0.001 |
| Alcoholism | 1562 | 2.1% | 859 | 2.5% | 0.001 | 72 | 1.1% | <0.001 |
| Baseline Medication Use | | | | | | | | |
| ACE/ARB | 42,490 | 58.0% | 20,561 | 58.6% | 0.054 | 4110 | 60.9% | <0.001 |
| Amiodarone | 8158 | 11.1% | 3591 | 10.2% | <0.001 | 1033 | 15.3% | <0.001 |
| Beta blockers | 42,590 | 58.1% | 21,311 | 60.7% | <0.001 | 4154 | 61.6% | <0.001 |
| H_2_-receptor antagonist | 4109 | 5.6% | 1660 | 4.7% | <0.001 | 475 | 7.0% | <0.001 |
| Proton pump inhibitor | 19,568 | 26.7% | 9488 | 27.0% | 0.239 | 2274 | 33.7% | <0.001 |
| Statins | 42,117 | 57.4% | 19,561 | 55.7% | <0.001 | 4063 | 60.2% | <0.001 |
| Anti-platelets | 11,502 | 15.7% | 5280 | 15.0% | 0.006 | 1502 | 22.3% | <0.001 |
| NSAIDS | 15,466 | 21.1% | 8837 | 25.2% | <0.001 | 1444 | 21.4% | 0.553 |

ACE: angiotensin-converting enzyme inhibitor; ARB: angiotensin-receptor blocker; CHADS_2_: congestive heart failure, hypertension, age ≥75 years, diabetes mellitus, prior stroke or transient ischemic attack or thromboembolism; CHA_2_DS_2_-VAS_C_: congestive heart failure, hypertension, age ≥75 years, diabetes mellitus, prior stroke or transient ischemic attack or thromboembolism, vascular disease, age 65–74 years, sex category; CHF: congestive heart failure; HAS-BLED: hypertension, abnormal renal and liver function, stroke, bleeding, labile international normalized ratios, elderly, drugs and alcohol; NSAIDs: nonsteroidal anti-inflammatory drugs; SD: standard deviation; stroke/SE: stroke/systemic embolism; TIA: transischemic attack.

^†^As the international normalized ratio value is not available in the databases, a modified HAS-BLED score was calculated with a range of 0 to 8.
